# Protocol for CONSORT-SPI: an extension for social and psychological interventions

**DOI:** 10.1186/1748-5908-8-99

**Published:** 2013-09-02

**Authors:** Paul Montgomery, Sean Grant, Sally Hopewell, Geraldine Macdonald, David Moher, Susan Michie, Evan Mayo-Wilson

**Affiliations:** 1 Centre for Evidence-Based Intervention, University of Oxford, Barnett House, 32 Wellington Square, Oxford OX1 2ER, UK; 2 Centre for Statistics in Medicine, University of Oxford, Botnar Research Centre, Windmill Road, Oxford, OX3 7LD, UK; 3 Institute of Child Care Research, Queen’s University Belfast, 6 College Park, Belfast BT7 1LP, UK; 4 Clinical Epidemiology Program, Ottawa Hospital Research Institute, Centre for Practice-Changing Research (CPCR), The Ottawa Hospital - General Campus, 501 Smyth Rd, Room L1288, Ottawa, ON K1H 8L6, Canada; 5 Centre for Outcomes Research and Effectiveness, Research Department of Clinical, Educational & Health Psychology, University College London, 1-19 Torrington Place, London WC1E 7HB, UK

**Keywords:** CONSORT-SPI, Randomised controlled trial, RCT, Reporting guidelines, Complex interventions

## Abstract

**Background:**

Determining the effectiveness of social and psychological interventions is important for improving individual and population health. Such interventions are complex and, where possible, are best evaluated by randomised controlled trials (RCTs). The use of research findings in policy and practice decision making is hindered by poor reporting of RCTs. Poor reporting limits the ability to replicate interventions, synthesise evidence in systematic reviews, and utilise findings for evidence-based policy and practice. The lack of guidance for reporting the specific methodological features of complex intervention RCTs contributes to poor reporting. We aim to develop an extension of the Consolidated Standards of Reporting Trials Statement for Social and Psychological Interventions (CONSORT-SPI).

**Methods/design:**

This research project will be conducted in five phases. The first phase was the project launch, which consisted of the establishment of a Project Executive and International Advisory Group, and recruitment of journal editors and the CONSORT Group. The second phase involves a Delphi process that will generate a list of possible items to include in the CONSORT Extension. Next, there will be a formal consensus meeting to select the reporting items to add to, or modify for, the CONSORT-SPI Extension. Fourth, guideline documents will be written, including an explanation and elaboration (E&E) document that will provide detailed advice for each item and examples of good reporting. The final phase will comprise guideline dissemination, with simultaneous publication and endorsement of the guideline in multiple journals, endorsement by funding agencies, presentations at conferences and other meetings, and a dedicated website that will facilitate feedback about the guideline.

**Conclusion:**

As demonstrated by previous CONSORT guidelines, the development of an evidence-based reporting guideline for social and psychological intervention RCTs should improve the accuracy, comprehensiveness, and transparency of study reports. This, in turn, promises to improve the critical appraisal of research and its use in policy and practice decision making. We invite readers to participate in the project by visiting our website (http://tinyurl.com/CONSORT-study).

## Background

Social and psychological interventions that aim to improve health and related outcomes are often complex and challenging to evaluate. As outlined in the Medical Research Council (MRC, UK) Framework for developing and evaluating complex interventions [1], they usually have multiple, interacting components at several levels, and may have multiple and variable outcomes that require sophisticated assessments and analyses. Randomised controlled trials (RCTs) provide the least biased estimates of effectiveness despite these complexities [2]. When reported clearly and completely, RCTs can be appropriately included in systematic reviews and practice guidelines, leading to better routine service and policy-related outcomes. When detailed information about study conduct is poorly reported, or not reported at all, the link between research and practice is weakened, and scarce resources are wasted [3]. Thus, to have its intended impact, the methods for reporting trials must be as rigorous as those for conducting them.

### Reporting guidelines

Reporting guidelines do not prescribe research conduct; they suggest those items of information that are necessary to understand how a study was conducted. The most widely-cited reporting guideline is the Consolidated Standards of Reporting Trials (CONSORT) Statement. Its main checklist has 25-items for reporting two-group parallel RCTs [4], and extension guidelines address other types of medical RCTs, such as cluster [5], pragmatic [6], and non-pharmacological intervention trials [7]. Opinion leaders and decision makers made rigorous use of empirical evidence and consensus development techniques to inform the content of CONSORT and its extension guidelines [8]. Since their publication, the reporting of thousands of medical RCTs have improved [9], with reports published in journals endorsing CONSORT improving more than those in other journals [10,11].

### A new guideline for social and psychological intervention trials

Despite improvements in the reporting of RCTs in medical disciplines, several studies indicate that the reporting quality of RCTs in the social and behavioural sciences remains suboptimal [12-16]. We conducted a systematic review of reporting guidelines for social and psychological intervention RCTs, as well as the quality of current reports of these studies. This review concluded that existing guidelines lacked the required rigour in their development, they have important limitations in their included reporting guidance, and they are poorly disseminated. Furthermore, most leading journals in these disciplines do not ask authors to follow any reporting guides, and important details are routinely missing from publications of social and psychological intervention RCTs [17].

To address these issues, many researchers and journal editors have proposed amending the CONSORT Statement to address these important complexities of social and psychological interventions and of their evaluation [18-23]. A new CONSORT extension developed by drawing on previous reporting guidance, up-to-date scientific literature, and stakeholder involvement and insight, could significantly improve the reporting of social and psychological intervention RCTs. This paper describes the project plan for a new guideline—CONSORT-SPI—which will include a checklist of reporting items and a participant flowchart that offer authors recommendations to accurately, comprehensively, and transparently describe these studies.

## Methods/design

**Figure 1:**
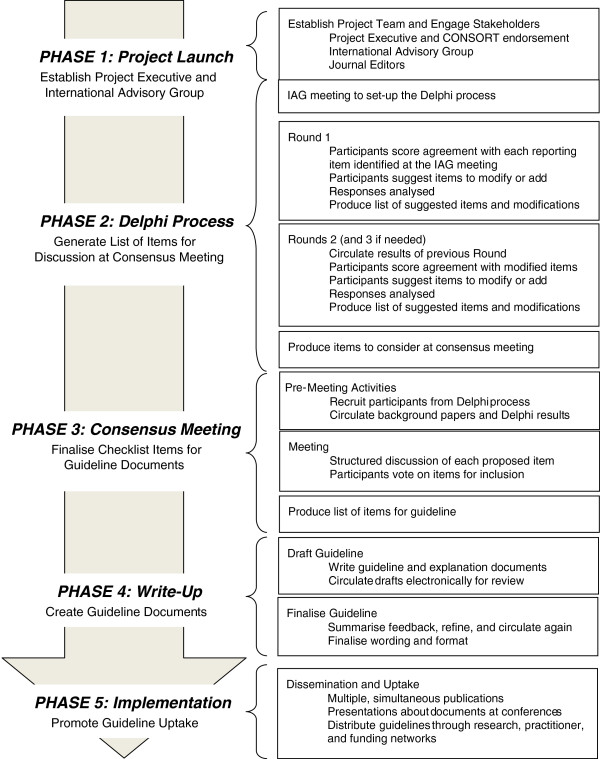
Workflow for CONSORT-SPI: an extension for social and psychological interventions.

The methods will follow recommended techniques for developing and disseminating reporting guidelines [9,24]. Aspects of these methods have been previously used to develop the CONSORT statement [4] and its extensions [25-28]; the SPIRIT statement for trial protocols [29]; and other guidelines [5,6]. This earlier work suggests the project will take 20 to 24 months to complete [24]. The project will involve five phases: the project launch, a Delphi process, a consensus development conference, writing up the guideline documents, and guideline implementation (see Figure 1).

### Phase one: project launch

To ensure project success, the following have already been secured for the launch of the project: a project executive; an International Advisory Group (IAG) of key opinion leaders across core fields; participation from high impact-factor journal editors for the consensus process; agreement from and collaboration with the CONSORT Group; and funding to support guideline development and dissemination.

### Project executive

The project executive (PM, EMW, SG, SM, GM, SH, and DM) has developed the project protocol and secured funding to complete the project, and are assembling the IAG and recruiting various stakeholders (*e.g.*, trialists, methodologists, practitioners, policy makers, funders, and recipients of services). The project executive will also run future phases of the project, including the Delphi process, consensus meeting, write-up of the resultant guideline documents, and the implementation strategy.

### International advisory group (IAG)

This team of key opinion leaders will advise at each project stage, and will help draft and disseminate the final guideline documents. Members are leading experts in social and psychological interventions across various disciplines (see Acknowledgments). They will help recruit stakeholders to participate in the project and identify topics to discuss at each stage. They will aid dissemination by endorsing and using the guideline, and by presenting it to relevant stakeholders in their respective fields.

### Journal editors

The most widely used reporting guidelines have enlisted journal editors during development and acquired official journal endorsement upon completion [9]. To begin this effort, editors of high impact-factor journals in key disciplines have been approached, and many have already agreed to participate. We encourage any other journal editors interested in participating in the project to contact us [30-37].

### CONSORT group

To increase successful uptake, new reporting guidelines for RCTs should be officially related to the CONSORT Statement. Previous reviews have found no high impact-factor journal that explicitly recommends an RCT reporting guideline other than the CONSORT statement [38]. Members of the CONSORT Group (DM and SH) are involved in this project, and the resultant guideline will be an official extension of the CONSORT Statement. The CONSORT Group’s success, collective experience, and prominence in the reporting guideline field—including the more recent SPIRIT guidelines for reporting protocols of trials [29]—will help to ensure the use of proper methods for developing and disseminating a high-quality reporting guideline.

### Phase two: the Delphi process

The purpose of phase two is to identify those areas in the reporting of social and psychological intervention trials that are most important for inclusion in the guideline. To involve a wide range of participants at this phase, an online, modified Delphi process will be conducted. The Delphi process will consist of a series of structured questionnaires completed anonymously by expert participants. Summarised responses from each questionnaire will be returned to the participants after each round, along with a new questionnaire to answer, until consensus is reached [39]. This process will help address areas of uncertainty, and measure and reach consensus [40].

### IAG meeting

The IAG will meet before round one of the Delphi process to nominate items for the initial questionnaire and to suggest credible participants for the process [39]. Prior to the meeting, the IAG will receive literature reviews regarding previous reporting guidelines for social and psychological intervention RCTs and their reporting quality, together with feedback from a consultation held at the 2012 Cochrane Colloquium [41]. These literature reviews will be used to generate items for the Delphi round one survey. Procedures for data collection, data analysis, and cut-offs for consensus [42] will be decided in light of recommended techniques for guideline development [9], and previous Delphi processes used to develop reporting guidelines [27,43].

### Recruitment

To enhance credibility and ensure widespread acceptance, the project will recruit informed and interested participants representing stakeholders that the guideline is intended to influence [42]. The IAG will help identify an initial list of stakeholders who extensively publish, fund, or utilise social and psychological intervention research, and a ‘snowball recruitment’ approach will be used via collaborators in relevant research and professional networks [4,12]. In order to engage those who might not be identified through snowball recruitment, the project website enables stakeholders to register their interest in participating. In addition, a commentary written by the study team and co-published in several journals, invites other stakeholders to participate [30-37]. Intervention researchers, methodologists, and guideline developers will form a substantial number of the participants [9]. Editors of high impact-factor journals will be invited for their expertise and to ensure uptake upon completion [44] Funders of social and psychological intervention studies will be invited to provide expertise and to promote use of the guideline for assessing grant applications [6]. Practitioners will help identify issues of relevance to practice [45]. Policy makers will help identify items, and they will assist in the creation of a user-friendly document and standards [24,46]. Representatives from consumer groups will advance the relevance of research reports to the ultimate recipients of services.

### Structure

Identified stakeholders will be invited to participate in an online Delphi survey to nominate checklist items for the CONSORT-SPI Extension. In each round, participants will be asked to rate (on a 1 to 10 Likert scale) the importance of including proposed checklist items, explain the reasons for their ratings, make suggestions for modifications, and indicate any missing items that should be considered. We expect the IAG to propose 25 to 70 checklist items in round one [7,43]. Items in later rounds will be based on responses from each previous round, and participants will receive summaries of quantitative and qualitative responses from the previous round to inform their new rankings [47]. At the end of the Delphi process, high-ranking items will be proposed for inclusion in the checklist during the consensus meeting. Low-ranking items will not be considered at the consensus meeting unless the project executive identifies valuable issues to discuss. Middle-ranking items will be discussed at the consensus meeting for possible inclusion or exclusion. We estimate that two to three rounds will be needed to obtain consensus, and that each round will take 30 to 45 minutes to complete [47].

### Phase three: consensus development conference

The purpose of phase three is to select the specific reporting items to be included in the new guideline. A consensus development conference will be held to determine guideline content, rather than wording or format [40]. This time has been allotted to allow sufficient time for thorough discussion, reducing hasty decision making that can hinder judgment [42].

### Participants

Participants will be recruited by discipline from the Delphi process by the project executive and the IAG, to include a range of stakeholder perspectives [8]. The size of the group (20 to 30 participants) will balance diversity of opinion with opportunities for interaction [9].

### Structure

The consensus meeting will follow methods [40] used in previous CONSORT meetings [4,6,25,27]. Literature reviews and the results of the Delphi process will be provided to participants in advance, and the conference will include background presentations [27], to ground conversations on empirical information and to facilitate cohesive discussion [42]. Participants will be led in structured discussions of, and vote on, each item proposed for the checklist from the Delphi process [27]. Care will be taken to ensure that all participants express views, that all ideas are discussed in-depth, and that assertive participants do not dominate the discussion [42]. Voting will be confidential using anonymous ballots to promote honest answers and allow participants to rethink their position if a re-vote is needed [47]. The meeting will conclude with discussion about optimising dissemination, and members of the group will commit to specific efforts to this end [27].

### Phase four: drafting the guideline documents

The purpose of this phase is to draft the guideline documents so that their wording and content is clear, precise, and suitable for all relevant disciplines.

### Draft guidelines and elaboration document

Following the consensus meeting, the proposed checklist will be reviewed by the project executive. The first goal will be to draft a checklist using concise, unambiguous, yet comprehensive wording. Each item will be supported with empirical evidence of previous poor reporting and implications for internal and external validity. In addition to the guideline statement, an Explanation and Elaboration (E&E) Document will explain in-depth the scientific rationale for each recommendation and provide an example of clear reporting for each item. This additional document will help editors and authors understand the importance of these guidelines, students and researchers understand the relevant issues, and authors meet the guideline requirements [9].

### Feedback

Drafts of the checklist will be circulated to consensus group participants to check that the documents accurately represent the decisions made during the meeting, provide examples of good reporting for specific items, and are useful for their intended purpose [27]. Feedback is important to evaluate the validity of consensus methods [42]. Responses will be incorporated into a statement that reports the project rationale, process methodology, and final included reporting items.

### Phase five: guideline implementation

The goal of the dissemination plan is to maximise awareness, understanding, and use of the CONSORT Extension when reporting social and psychological intervention trials.

### Dissemination methods

The dissemination strategy includes stakeholder involvement in the design and execution of this project, ensuring that the guideline will be acceptable and widely endorsed. Next, simultaneous publications in multiple, high impact-factor journals will begin the process of dissemination and uptake [24]. The IAG will identify and approach the most appropriate journals in key disciplines to publish the guideline and provide an editorial supporting the guideline. The IAG will ask editors from all relevant journals to endorse the guideline. Endorsement will involve clear directions in each journal’s ‘Instructions to Authors’ that the guideline should be followed and the checklist should be included in all relevant submissions [24,36].

Open-access publications are key to widespread uptake of the reporting guideline [44]. Our intention is to seek instant open access publications, allowing us to retain ownership of the work to facilitate broad dissemination. We will also make the guideline, and other relevant documents, including the E&E document, available on our website as well as other websites (*e.g.*, the CONSORT Group, the EQUATOR Network for reporting guidelines). A dedicated webpage will be used to discuss new, relevant evidence related to social and psychological intervention trials, and to ask the wider scientific community to provide feedback on their experiences of using the guideline, in order to allow for the guideline’s continual development [48]. The project executive and IAG will present the guideline at influential conferences, professional bodies, and organisations within their respective fields.

## Conclusion

These methods were chosen to develop the best reporting standards, generate consensus, and promote widespread dissemination and uptake of CONSORT-SPI. They are based on best practice and evidence-based principles. Research-informed purposive sampling by the IAG will provide a less biased selection of participants than the project executive could provide alone [42]; the involvement of various stakeholders in guideline development will ensure that a variety of perspectives are captured. The resulting multidisciplinary, international consensus will maximise the impact of the guideline beyond any specialist field [40]. Moreover, formal consensus development methods are increasingly employed in guideline development, especially when evidence (and opinion) are contradictory or insufficient [39]. These techniques capture the advantages of group decision-making while overcoming biases associated with less structured group methods [42]. Previous research suggests that these methods are the most appropriate for our purposes [40], and are beneficial to use in combination [49]. For example, the online Delphi process is a cost-effective way to involve a large number of international and cross-disciplinary participants [47], and it has been successful in previous guidelines [4,6,11,13].

If executed successfully, the outputs from this project will help authors write clear reports, create a framework for reviewers to assess publications, expedite funding evaluations, provide a pedagogical tool for training students and researchers in trial methodology, and help research consumers evaluate RCT validity and applicability [50,51]. In these ways, the guideline aims to improve the reporting quality of social and psychological intervention RCTs and facilitate the efficient, effective transfer of research evidence into real-world use. We invite readers to participate in the project by visiting our website (http://tinyurl.com/CONSORT-study).

### Project website

For more on the CONSORT extension, see http://www.tinyurl.com/CONSORT-study.

### Ethics

The conduct of this research project will conform to the appropriate ethical and legal standards regarding informed consent, confidentiality, and data storage. Ethics approval was obtained from the Department Research Ethics Committee (DREC) for the Department of Social and Intervention, University of Oxford (Ref: 2011-12_83).

### Data preservation

We commit to the long-term preservation and availability for use by other research teams of the high-quality data produced by this project. The data will be prepared to allow independent usage. The Centre for Evidence Based Intervention (CEBI) at Oxford University is well placed to host this work. It has full University support for this project and the CONSORT Group is close at hand to assist where needed. All data will be safely stored and backed-up at CEBI.

## Competing interests

The authors declare that they have no competing interest.

## Authors’ contributions

PM, EMW, and SG conceived of the idea for the project. All authors helped to draft the manuscript, and all have read and approved the final manuscript.
